# Sub-MICs of *Mentha piperita* essential oil and menthol inhibits AHL mediated quorum sensing and biofilm of Gram-negative bacteria

**DOI:** 10.3389/fmicb.2015.00420

**Published:** 2015-05-13

**Authors:** Fohad M. Husain, Iqbal Ahmad, Mohammad S. Khan, Ejaz Ahmad, Qudisa Tahseen, Mohd Shahnawaz Khan, Nasser A. Alshabib

**Affiliations:** ^1^Department of Agricultural Microbiology, Aligarh Muslim UniversityAligarh, India; ^2^Department of Food Science and Nutrition, College of Food and Agricultural Sciences, King Saud UniversityRiyadh, Saudi Arabia; ^3^School of Pharmaceutical Sciences, Sao Paulo State UniversityAraraquara, Brazil; ^4^Department of Zoology, Aligarh Muslim UniversityAligarh, India; ^5^Department of Biochemistry, Protein Research Chair, College of Science, King Saud UniversityRiyadh, Saudi Arabia

**Keywords:** anti-quorum sensing activity, peppermint oil, menthol, biofilm, molecular docking, *C. elegans*

## Abstract

Bacterial quorum sensing (QS) is a density dependent communication system that regulates the expression of certain genes including production of virulence factors in many pathogens. Bioactive plant extract/compounds inhibiting QS regulated gene expression may be a potential candidate as antipathogenic drug. In this study anti-QS activity of peppermint (*Mentha piperita*) oil was first tested using the *Chromobacterium violaceum* CVO26 biosensor. Further, the findings of the present investigation revealed that peppermint oil (PMO) at sub-Minimum Inhibitory Concentrations (sub-MICs) strongly interfered with acyl homoserine lactone (AHL) regulated virulence factors and biofilm formation in *Pseudomonas aeruginosa* and *Aeromonas hydrophila*. The result of molecular docking analysis attributed the QS inhibitory activity exhibited by PMO to menthol. Assessment of ability of menthol to interfere with QS systems of various Gram-negative pathogens comprising diverse AHL molecules revealed that it reduced the AHL dependent production of violacein, virulence factors, and biofilm formation indicating broad-spectrum anti-QS activity. Using two *Escherichia coli* biosensors, MG4/pKDT17 and pEAL08-2, we also confirmed that menthol inhibited both the *las* and *pqs* QS systems. Further, findings of the *in vivo* studies with menthol on nematode model *Caenorhabditis elegans* showed significantly enhanced survival of the nematode. Our data identified menthol as a novel broad spectrum QS inhibitor.

## Introduction

Emergence and spread of antibiotic resistance among pathogenic bacteria represents a major obstacle in treatment of infectious diseases. About 80% of the infections caused by microorganisms are biofilm based (Davies, 2003). Biofilm architecture consists of structured and aggregated communities of bacteria encased in a self-secreted exopolymeric substance (EPS; Costerton et al., 1995). Several studies have revealed that bacteria have developed resistance because of the prolonged treatment with conventional antibiotics possessing a broad-range efficacy via toxic or growth-inhibitory effects on target organisms rendering the traditional antibiotic treatment virtually ineffective (Xavier and Bassler, 2003). It has been found that bacteria living in the biofilm mode of growth are resistant to antibiotic up to 1000 times more than their planktonic counterparts (Caraher et al., 2007). Therefore, there is an increased demand for developing alternative strategies to the conventional antibiotic therapy (Zeng et al., 2008). Bacterial quorum sensing (QS) has been identified as a promising anti-infective drug target (Hentzer et al., 2002). It would be possible to repress the expression of QS regulated phenotypes through which the development of biofilm and virulence are being accomplished in many Gram-negative bacterial pathogens; such interference is expected to be useful in the treatment of bacterial infections (Hentzer and Givskov, 2003; March and Bentley, 2004). The QS mechanism enables bacteria to detect their population density through the production, release, and perception of small diffusible molecules called autoinducers and to coordinate gene expression accordingly (Williams et al., 2007; Rumbaugh et al., 2012).A wide array of functions in bacteria ranging from bacterial cell motility to complex behaviors such as biofilm formation and production of virulence factors are regulated by QS in pathogenic bacteria (Atkinson et al., 2006; Rumbaugh and Armstrong, 2014). Several Gram-negative pathogens employ *N*-acyl homoserine lactones (AHLs)-mediated QS systems to coordinate and synchronize specific gene expression of particular phenotypic features between the individual cells (Fuqua et al., 1994).

In *Pseudomonas aeruginosa*, biofilm formation and the production of various virulence factors are regulated via the action of a hierarchical quorum-sensing system mediated by the two chemically different classes of signal molecules, the *N*-acylhomoserine lactones and the 4-quinolones which comprise of more than 50 compounds and includes the most active signal molecule 2-heptyl-3-hydroxy-4-quinolone which is popularly known as the pseudomonas quinolone signal (PQS; Häussler and Becker, 2008; Heeb et al., 2011).

Quorum sensing plays an important role during the initial event of infection for the common opportunistic Gram-negative human pathogen *P. aeruginosa*, which is associated with nosocomial and wound infections, immunocompromised (Obritsch et al., 2005; LaSarre and Federle, 2013) and the genetically inherited disease cystic fibrosis (Fothergill et al., 2007). Therefore, compounds that interfere with the QS system to attenuate bacterial pathogenicity are termed as anti-QS compounds. Such compounds neither kill the bacteria nor stop their growth and are less expected to develop resistance toward antibiotics.

Various compounds both synthetic and natural from algae, fungi, and other organisms have been reported with quorum sensing inhibitory (QSI) activity, but they have little or no therapeutic value due to their unstable nature or toxicity associated with them (Rasmussen and Givskov, 2006). Therefore efforts have been directed in search of safe and effective QSI compounds from natural products particularly from medicinal plants, edible vegetables, spices and fruits, marine sponges and seaweeds (Zahin et al., 2010; Husain and Ahmad, 2013; Kalia, 2013).

Previously, we had screened 21 commonly available essential oils for their anti-QS activity using biosensor strains, *Chromobacterium violaceum* CV12472 and CVO26. The anti-QS activity of four essential oils namely cinnamon, lavender, clove, and peppermint was detected. Interestingly, most promising anti-QS activity against both *C. violaceum* pigment production and swarming motility of *P. aeruginosa* was demonstrated by clove oil at sub-Minimum Inhibitory Concentration (sub-MIC; Khan et al., 2009). Similarly, inhibition of QS signals by oils of rose, geranium, rosemary, and lavender has also been reported (Szabo et al., 2010; Yap et al., 2014).

Considering the encouraging results of the primary screening on peppermint oil (PMO; Hi-media, Mumbai, India), we have investigated its anti-pathogenic potential and its major phytoconstituent menthol (Hi-media, Mumbai, India), in three test organisms (*C. violaceum*, *P. aeruginosa,* and *Aeromonas hydrophila*). As above test strains use one or more different types of autoindiceer molecules activity all three organism may provide a strong basis for selection of broad spectrum anti-QS compounds. Both PMO and its major constituent menthol were studied for their efficacy *in vitro*. Further efficacy of major active component, Menthol was evaluated in *Caenorhabditis elegans* model to uncover the therapeutic potential of menthol as effective QS inhibitor.

## Materials and Methods

### Bacterial Strains and Growth Conditions

Bacterial strains used in this study were *Chromobacterium violaceum* CV026 (a mini-Tn5 mutant of *C. violaceum* 31532 that cannot synthesize its own AHL but responds to exogenous C4 and C6 AHLs), *P. aeruginosa* PAO1 (C4 and 3-oxo-C12 HSL producer, McLean et al., 2004), *Escherichia coli* pEAL08-2 (*E. coli* DH5α harboring plasmid pEAL08-2, Cugini et al., 2007), and *E. coli* MG4/pKDT17 (*E. coli* DH5α harboring plasmid pMG4/pKDT, Pearson et al., 1994). All the bacterial strains were grown in Luria-Bertani (LB) medium at 30°C for 24 h. When required, the medium for *C. violaceum* CV026 was supplemented with hexanoyl homoserine lactone (C6-HSL; Sigma–Aldrich, St Louis, MO, USA).

### Determination of Minimum Inhibitory Concentration

Minimum Inhibitory Concentration of the test agents were determined against *C. violaceum* CVO26, *P. aeruginosa* PAO1, and *A. hydrophila* WAF38 by broth macrodilution method (Clinical and Laboratory Standards Institute [CLSI], 2007). Sub-MICs were selected for the assessment of anti-virulence and anti-biofilm activity in the above test strains.

### Quantitative Estimation of Violacein

Extent of violacein production by *C. violaceum* (CVO26) in the presence and absence of Sub-MICs of test agents was studied by extracting violacein and quantifying photometrically using method of Blosser and Gray (2000) with little modifications (Packiavathy et al., 2012). Briefly, 1-ml culture from each flask was centrifuged at 13000 rev/min for 10 min to precipitate the insoluble violacein. The culture supernatant was discarded and 1 ml of DMSO was added to the pellet. The solution was vortexes vigorously for 30 s to completely solubilize violacein and centrifuged at 13000 rev/min for 10 min to remove the cells. Two hundred microlitres of the violacein-containing supernatants were added to 96-well flat bottomed microplates (Polylab, India), four wells per each solution, and the absorbance was read with a microplate reader (Thermo Scientific Multiskan Ex, India) at a wavelength of 585 nm. Reduction in the production of pigment in the presence of test agents was measured in terms of % inhibition as, [(OD of control – OD of treated)/OD of control] × 100.

### Effect on Virulence Factor Production

Effect of sub-MICs of PMO and menthol on virulence factors of *P. aeruginosa* and *A. hyrophila* such as LasB elastase, protease, pyocyanin, chitinase, swarming motility, EPS extraction, and quantification was assessed as described previously (Husain et al., 2013).

### Assay for Biofilm Inhibition

The effect of PMO and menthol on biofilm formation was measured using the microtitre plate assay (O’Toole and Kolter, 1998). Briefly, 1% overnight cultures (0.4 OD at 600 nm) of test pathogens were added into 1 mL of fresh LB medium in the presence and the absence of sub-MICs of test agents. Bacteria were allowed to adhere and grow without agitation for 24 h at 30°C. After incubation, microtitre plate was emptied by removing the media along with free-floating planktonic cells and the wells were gently rinsed twice with sterile water. The surface-attached cells (biofilm) were stained with 200 μL of 0.1% crystal violet (CV; Hi-media, Mumbai, India) solution. After 15 min, CV solution was discarded completely and wells were filled with 200 μL of 95% ethanol to solubilize CV from the stained cells. The biofilm biomass was then quantified by measuring the absorbance at OD 470 nm in a microplate reader (Thermo Scientific Multiskan Ex, India).

### Scanning Electron Microscopy

Biofilms were grown on glass coverslips, in the treated and untreated cultures of PAO1. After 24 h incubation, the cover slips were rinsed with distilled water to remove planktonic cells and processed for scanning electron microscopy (SEM) examination as described by Husain et al. (2013).

### *Caenorhabditis elegans* Survival Assay

The method described by Musthafa et al. (2012) was adopted to study the *in vivo* efficiency of sub-MIC of menthol in *Caenorhabditis elegans* here nematode model. Briefly, the young adult nematodes were infected with PAO1 in the 24-well microtitre plate and incubated at 25°C for 12 h. After incubation, *C. elegans* from the wells were washed thrice with M9 buffer to remove surface-bound bacteria. Around 10 infected worms were transferred to the wells of microtitre plate containing 10% LB broth in M9 buffer and incubated at 25°C with or without 800 μg/mL menthol treatment. The plate was scored for live and dead worms every 12 h for 4 days. To assess the toxicity if any of the oil, *C. elegans* with menthol was maintained.

### GC–MS Analysis

The compositions of the PMO was analyzed by Perkin Elmer GC Autosystem XL and Turbomass with EI source using PE-Wax column (30 m × 0.25 mm i.d., film thickness 0.25 mm), carrier gas was helium with column head pressure 7 psi connected to data station. Temperature programming: 4 min at 60°C then using at 4°C min^-1^ to 200°C with hold time of 21 min, at 200°C, split ratio 1:50. The components were identified by comparing their retention times to those of authentic samples, as well as by comparing their mass spectra with those of Wiley 8 and NIST 05 Libraries described by Masada (1976). Quantitative data were obtained by the peak normalization technique using integrated FID response.

### Molecular Docking Analysis

The protein data bank (PDB) structure 2UV0 of LasR was downloaded from Brookhaven Protein Databank for molecular docking of phytoconstituents obtained from *Mentha piperita* essential oil to natural autoinducer AHL binding site of LasR. The residues falling within 5 Å of the binding site were extracted and combined to define the binding site residues. From Pubchem database the SDF format for 3D structures of all the phytoconstituents Limonene (I); Menthone (II); Isomenthone (III); 1-Hydroxyoctane (IV); Isopulegol (V); Menthyl acetate (VI); Neoisomenthol (VII); 2-Isopropyl-5-methylcyclohexanol (VIII); Menthol (IX); Lavandulol (X); Piperitone (XI) as well as the natural autoinducer 3-oxo-C12-HSL or AHL (0) with their chemical identifier (CID) 22311; 26447; 6986; 957; 24585; 27867; 19244; 1254; 165675; 5464156; 6987; and 3246941, respectively, were downloaded. The whole LasR molecule was defined as the binding site residues. Molecular docking simulation of the phytoconstituents (inhibitors) to LasR was performed with Autodock Vina program (Trott and Olson, 2010). Autodock uses Lamarkian genetic algorithm to calculate the possible conformations of the ligand that binds to the protein. Here the flexible ligands were set to free in the binding clefts of LasR to dock in the most feasible way. The grid centers of 24.327, 11.9665, and 77.3765 with the grid sizes of 40.368, 46.223, and 56.579 for X, Y, and Z axes, respectively, were used for covering all the binding site residues. For docking simulations the exhaustiveness was set to 8. Best docked structures based on the binding energy scores (ΔG) were chosen for further analyses. The hydrogen bonding and hydrophobic interactions between ligand and protein were calculated by Accelrys DS Visualizer 2.0 (Accelrys Software Inc., 2012) while the **Figure 2** were generated by PyMol version 0.99 (DeLano, 2002) and Accelrys DS Visualizer 2.0 (Accelrys Software Inc., 2012). To validate the LasR-inhibitor docking experiments, we have compared our docking results with the structures already available at the PDB where the the natural autoinducer AHL has been co-crystallized with LasR, as for example, 2UV0 (BottomLey et al., 2007). Here, all the ligands from the crystal structure have been removed and the AHL was re-docked.

The binding constants (K_b_) for protein-ligand interactions were calculated from the obtained free energy changes of docking by using the following equation:

$$\Delta G=-RT\ln K_{b}$$

where R is the gas constant (1.987 cal.mol^-1^.K^-1^) and T is 298 K (Ahmad et al., 2011).

### Analysis of *las*B and *pqs*A Transcriptional Activity in *E. coli*

Measurements of β-galactosidase luminescence in *E. coli* MG4/pKDT17 and *E. coli* pEAL08-2 was done by the adopting the method described by Pearson et al. (1994) and Cugini et al. (2007).

### Statistical Analysis

All experiments were performed in triplicates and the data obtained from experiments were presented as mean values and the difference between control and test were analyzed using student’s *t*-test.

## Results

### Minimum Inhibitory Concentration

Minimum inhibitory concentration of PMO was determined to select the sub-MICs to study the effect on growth and inhibition of QS regulated functions. MIC of oil was found to be 0.6% v/v against *C. violaceum* CVO26, 6.4% v/v against *P. aeruginosa* PAO1, and 1.6% v/v against *A. hydrophila* WAF38. The MIC of menthol was found to be 1 mg/mL, 800 μg/mL, and 400 μg/mL against PAO1, CVO26, and WAF 38, respectively.

### Effect of Peppermint Oil on Violacein Production

**FIGURE 1:**
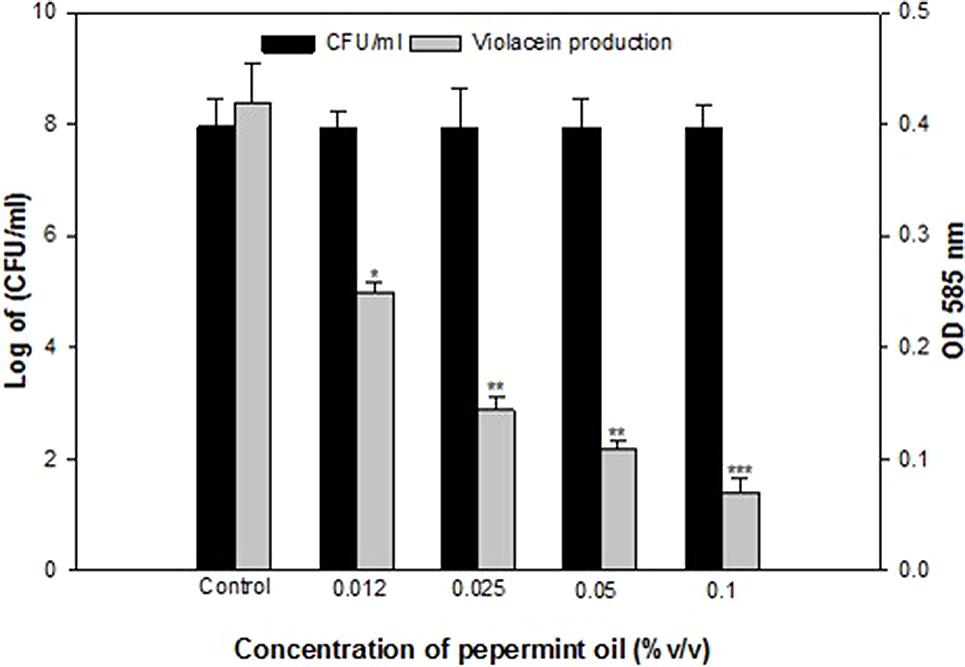
**Quantitative assessment of violacein inhibition in CVO26 by sub-MICs of peppermint oil (PMO).** All of the data are presented as mean ± SD. Significance at ^∗^*p* ≤ 0.05, significance at ^∗∗^*p* ≤ 0.005, significance at ^∗∗∗^*p* ≤ 0.001.

Anti-QS property of *M. piperita* (peppermint) oil was firstly assessed for pigment inhibition in CVO26. The result of the violacein quantification assay is depicted in **Figure 1**. The sub-MICs of PMO exhibited concentration-dependent violacein inhibitory activity at all tested concentrations and maximum reduction of 83.3% was recorded. Viable plate count performed on MHA plates at 24 h incubation showed no significant difference in the number of colony-forming units (CFUs) between untreated *C. violaceum* CVO26 and *C*. *violaceum* CVO26 treated with sub-MICs of the oil (**Figure 1**).

### Effect OF PMO on Production of Virulence Factors and Biofilm Formation

The effect of sub-MICs of PMO in reducing the production of QS-dependent LasB Elastase, protease and chitinase in PAO1 is presented in **Table 1**. Significant decrease in LasB elastase activity was observed in the culture supernatant of PAO1 treated with sub-MICs of PMO. A minimum of 30% inhibition was observed when PAO1 was cultured with oil at a concentration of 0.37% v/v and maximum of 80% inhibition was observed at 3% v/v oil concentration. The impact of PMO in inhibiting the QS-dependent protease activity of PAO1 was also determined and results of the obtained showed 26.6, 48.1, 70.4, and 76% decrease in total protease production when treated with 0.37, 0.75, 1.5, and 3% v/v oil concentrations, respectively. Treatment of PAO1 with sub-MICs of PMO showed significantly reduced chitinase activity, the oil (0.3–3% v/v) demonstrated inhibition in chitinase production to the level of 21–78% (**Table 1**).

**Table 1:** Effect of sub-MICs of peppermint oil (PMO) on inhibition of quorum sensing regulated virulence factors in *Pseudomonas aeruginosa* PAO1.

Concentration (% v/v)	Elastase activity^a^	Total protease^b^	Pyocyanin production^c^	Chitinase activity^d^	Exopolymeric substance (EPS) production^e^	Swarming motility^f^	Biofilm formation^g^
Control	0.145 ± 0.009	1.169 ± 0.034	6.1 ± 0.36	0.153 ± 0.011	1.278 ± 0.020	75 ± 2.7	0.406 ± 0.034
0.375	0.101 ± 0.016 (30.3)	0.857 ± 0.019 (26.6)	2.9 ± 0.30 (52.4)^∗∗^	0.120 ± 0.008 (21.5)	0.767 ± 0.013 (39.9)^∗^	37 ± 3.5 (50.6)^∗∗^	0.232 ± 0.021 (42.8)^∗^
0.75	0.075 ± 0.005 (48.2)^∗^	0.606 ± 0.010 (48.1)^∗^	2.3 ± 0.15 (62.2)^∗∗^	0.090 ± 0.013 (41.1)^∗^	0.501 ± 0.012 (59.2)^∗∗^	29 ± 2 (61.3)^∗∗^	0.156 ± 0.021 (61.5)^∗^
1.5	0.050 ± 0.008 (65.5)^∗∗^	0.345 ± 0.023 (70.4)^∗∗^	1.7 ± 0.25 (72.1)^∗∗^	0.054 ± 0.019 (64.7)^∗^	0.449 ± 0.010 (64.8)^∗∗^	22 ± 2 (70.6)^∗∗^	0.113 ± 0.017 (72.1)^∗∗^
3	0.029 ± 0.006 (80.0)^∗∗^	0.280 ± 0.013 (76.0)^∗∗^	0.9 ± 0.30 (85.2)^∗∗∗^	0.032 ± 0.006 (79.0)^∗∗^	0.3 ± 0.017 (76.52)^∗∗^	14 ± 1.5 (81.3)^∗∗∗^	0.064 ± 0.012 (84.2)^∗∗∗^

^a^Elastase activity is expressed as the absorbance at OD_495_.

^b^Total protease activity is expressed as the absorbance at OD_600._

^c^Pyocyanin concentrations were expressed as micrograms of pyocyanin produced per microgram of total protein.

^d^Chitinase activity is expressed as the absorbance at OD_570_.

^e^EPS production is expressed as absorbance at OD_480_.

^f^Swarming motility is expressed as diameter of swarm in mm.

^g^Biofilm formation is expressed as OD_470_ after incubation with crystal violet.

The data represents mean values of three independent experiments. Significance at ^∗^*p* ≤ 0.05, significance at ^∗∗^*p* ≤ 0.005, significance at ^∗∗∗^*p* ≤ 0.001. Values in the parentheses indicate percent reduction over control.

To examine the efficiency of the oil against pyocyanin production, the PAO1 cells were cultivated in the presence and absence of PMO. As a result, a significant decrease in pyocyanin production was observed, up to the level of 52.4–85.2%, respectively, in PAO1, at concentrations ranging from 0.3 to 3% v/v as compared to the control. The effect of PMO on swarming motility of PAO1 was also examined. The oil effectively inhibited QS dependent swarming migration in a dose dependent manner in *P. aeruginosa* PAO1. The maximum reduction of 81.3% in swarming migration was recorded at highest tested concentration (3% v/v) followed by 70.6, 61.3, and 50.6% at 1.5, 0.75, and 0.37% v/v oil concentration, respectively, (**Table 1**).

The spectrometric analysis of EPS extracted from oil treated and untreated cultures of PAO1 revealed that the production of EPS decreased with increasing concentration of PMO. The test oil at 3% v/v concentration exhibited 76% decrease in EPS production in *P. aeruginosa* PAO1. Anti-biofilm activity of the test oil shows biofilm inhibition of the pathogen. Addition of 0.37, 0.75, 1.5, and 3% v/v concentration of oil led to a dose dependent reduction in biofilm formation in the order of 42.7, 61, 73, and 84%, respectively, (**Table 1**).

Similarly, the oil of peppermint (0.1–0.8% v/v) effectively interfered with the QS system of *A. hydrophila* WAF38 by significantly reducing the total protease activity to the level of 24.5–71% (*p* ≤ 0.005) and EPS production by 39–77.9%. Maximum decrease (74.8%) in biofilm formation at 0.8% v/v concentration of the oil was observed as depicted in **Table 2**.

**Table 2:** Effect of sub-MICs of PMO on inhibition of quorum sensing regulated virulence factors in *Aeromonas hydrophila* WAF-38.

Concentration (% v/v)	Total protease^a^	EPS production^b^	Biofilm formation^c^
Control	0.847 ± 0.018	0.834 ± 0.038	0.310 ± 0.024
0.1	0.639 ± 0.034 (24.5)	0.508 ± 0.017 (39.0)	0.187 ± 0.014 (39.6)
0.2	0.413 ± 0.012 (51.2)^∗^	0.330 ± 0.011 (60.4)^∗^	0.143 ± 0.015 (53.8)^∗^
0.4	0.320 ± 0.013 (62.2)^∗^	0.238 ± 0.015 (71.4)^∗∗^	0.090 ± 0.021 (70.9)^∗∗^
0.8	0.245 ± 0.019 (71.0)^∗∗^	0.184 ± 0.006 (77.9)^∗∗∗^	0.078 ± 0.013 (74.8)^∗∗^

^a^Total protease activity is expressed as the absorbance at OD_600._

^b^EPS production is expressed as absorbance at OD_480_.

^c^Biofilm formation is expressed as OD_470_ after incubation with crystal violet.

The data represents mean values of three independent experiments. Significance at ^∗^*p* ≤ 0.05, significance at ^∗∗^*p* ≤ 0.005, significance at ^∗∗∗^*p* ≤ 0.001

Values in the parentheses indicate percent reduction over control.

### Effect of Peppermint Oil on *las* System

The addition of PMO decreased significant β-galactosidase activity in *E. coli* MG4/pKDT17 by up to 41.9 and 54.5% at 1.5 and 3% v/v (**Figure 5**), demonstrating that PMO reduces AHL levels significantly to inhibit las-controlled transcription.

### GC-MS Analysis of Peppermint Oil

Major ingredient of PMO as revealed by GC-MS analysis was menthol (36.87%), and other constituents identified were menthone (16.44%), neoisomenthol (11.33%), isomenthone (10.47%), menthyl acetate (7.47%), 2-isopropyl-5-methylcyclohexanol (2.74%), piperitone (2.17%), and limonene (0.53%) as given in Supplementary Table S1 and depicted in Supplementary Figure S1.

### Molecular Docking Analysis

**FIGURE 2:**
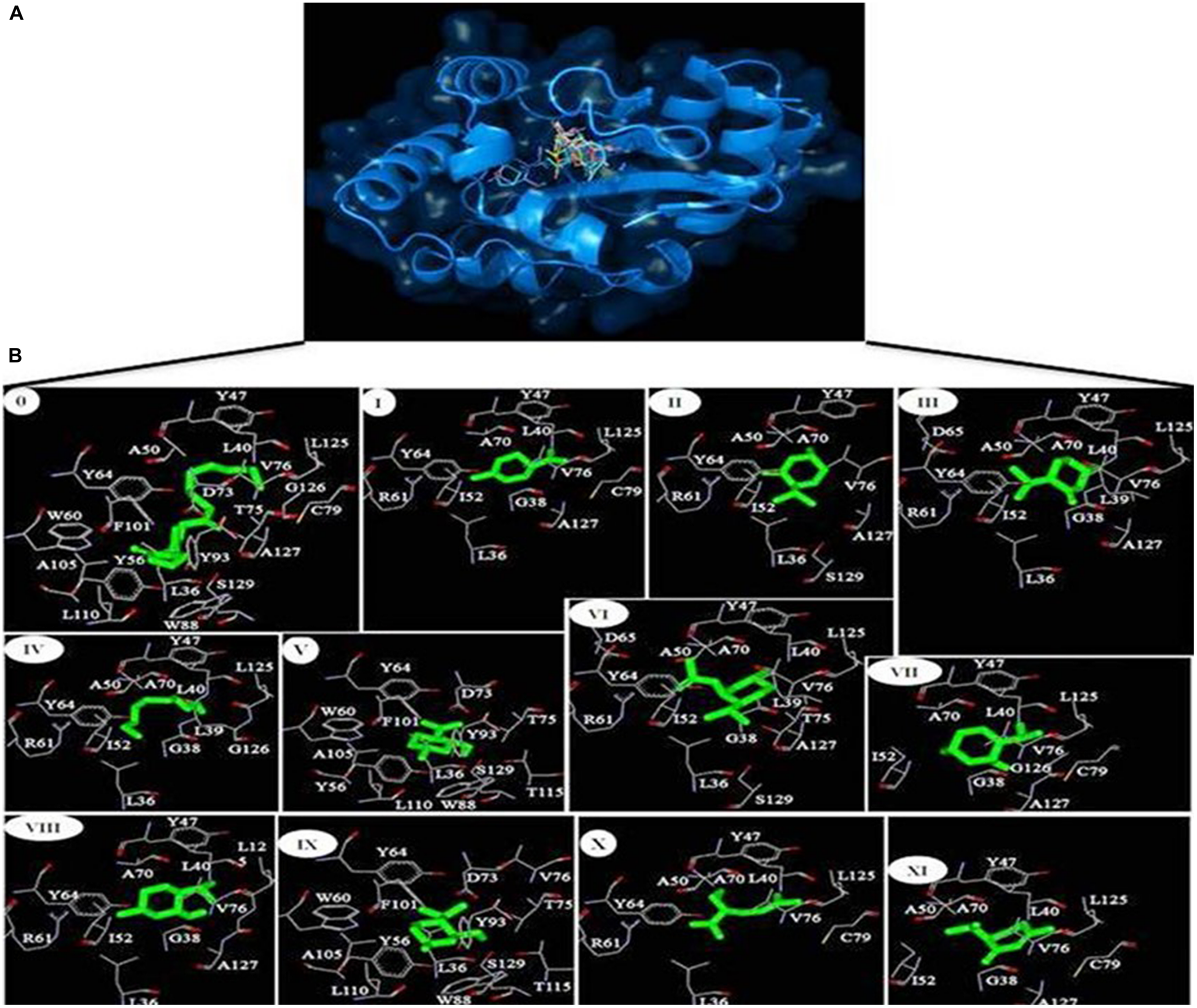
**(A)** Inhibitors obtained from the essential oil of *Mentha piperita* as well as the natural autoinducer 3-oxo-C12-HSL acyl homoserine lactone (AHL) are docked in the hydrophobic pocket of LasR. **(B)** Residues involved in the interaction of LasR-inhibitors as revealed from molecular docking 0, 3-oxo-C12-HSL; I, Limonene; II, Menthone; III, Isomenthone; IV, 1-Hydroxyoctane; V, Isopulegol; VI, Menthyl acetate; VII, Neoisomenthol; VIII, 2-Isopropyl-5-methylcyclohexanol; IX, Menthol; X, Lavandulol; XI, Piperitone.

For the better understanding of LasR-phytoconstituents binding the complementary applications of molecular docking of compounds on LasR has been performed with Autodock simulation analyses. The best energy ranked results are shown in **Figure 2** and are summarized in **Table 3**. As a reference model, the natural autoinducer AHL was docked in ligand binding domain of LasR. We found that L36, L40, Y47, A50, Y56, W60, Y64, D73, T75, V76, C79, W88, Y93, F101, A105, L110, L125, G126, A127, and S129 of LasR were interacted with AHL. Out of these, only Y56, W60, D73, and T75 formed hydrogen bonds whereas others were bound with hydrophobic interactions. In this way, the best scored docked AHL molecule occupied almost in the identical pattern as in the ligand-complexed crystal structure. This docked structure was further evaluated by superimposing with the LasR-AHL bound crystal structure and was found to be quite significant. From the above interacted residues Y47, A50, Y56, W60, Y64, D73, V76, and S129 are the main residues responsible for the activation of LasR. In case of all the 11 phytocompounds, most of the residues involved in the interactions are common with the natural ligand AHL (**Table 3**). Flexible ligands were set to free in the LasR to dock in the most feasible orientation according to the least free energy change (ΔG) and the interactions between the LasR and the inhibitors were exclusively hydrophobic in nature as reflected by several non-polar residues at binding site. These residues were in the proximity distance of 5 Å of the bound ligands. The essential oil constituents were docked in the active site with binding affinities ranging from -5.1 to -7.9 kcal/mol as compared to the binding affinity of AHL (-8.7 kcal/mol) and from these values the obtained binding constants (K_b_) were 5.50 × 10^3^ to 6.22 × 10^5^ as compared to the AHL (2.39 × 10^6^ M^-1^; **Table 3**). All the non-cyclic aliphatic compounds showed the lowest affinity while limonene was the weakest interacting cyclic entity. Here, most of the residues of LasR interacted hydrophobically with all the 11 tested compounds. Highest affinities are shown by menthol followed by isopulegol and 1-Hydroxyoctane as given in **Table 3**.

**Table 3:** Molecular docking results of LasR-inhibitor interactions.

Compounds^≈^	CID^∗^	K_b_ (M^-1^)	ΔG (kcal.mol^-1^)	Residues involved	Common residues^#^	H-bonds
0	3246941	2.39 × 10^6^	-8.7	20	–	4
I	22311	1.15 × 10^5^	-6.9	12	8	0
II	26447	1.36 × 10^5^	-7.0	10	7	1
III	6986	1.61 × 10^5^	-7.1	14	8	0
IV	957	5.50 × 10^3^	-5.1	12	7	1
V	24585	5.25 × 10^5^	-7.8	13	12	1
VI	27867	4.94 × 10^4^	-6.4	16	10	1
VII	19244	1.36 × 10^5^	-7.0	10	7	0
VIII	1254	3.16 × 10^5^	-7.5	12	8	0
IX	165675	6.22 × 10^5^	-7.9	14	13	2
X	5464156	3.52 × 10^4^	-6.2	10	8	0
XI	6987	1.90 × 10^5^	-7.2	10	7	0

^≈^0, 3-oxo-C12-HSL; I, Limonene; II, Menthone; III, Isomenthone; IV, 1-Hydroxyoctane; V, Isopulegol; VI, Menthyl acetate; VII, Neoisomenthol; VIII, 2-Isopropyl-5-methylcyclohexanol; IX, Menthol; X, Lavandulol; XI, Piperitone.

^∗^PubChem chemical identifier.

^#^With respect to autoinducer or natural inhibitor (3-oxo-C12-HSL).

### Effect of Menthol on Violacein Production

**FIGURE 3:**
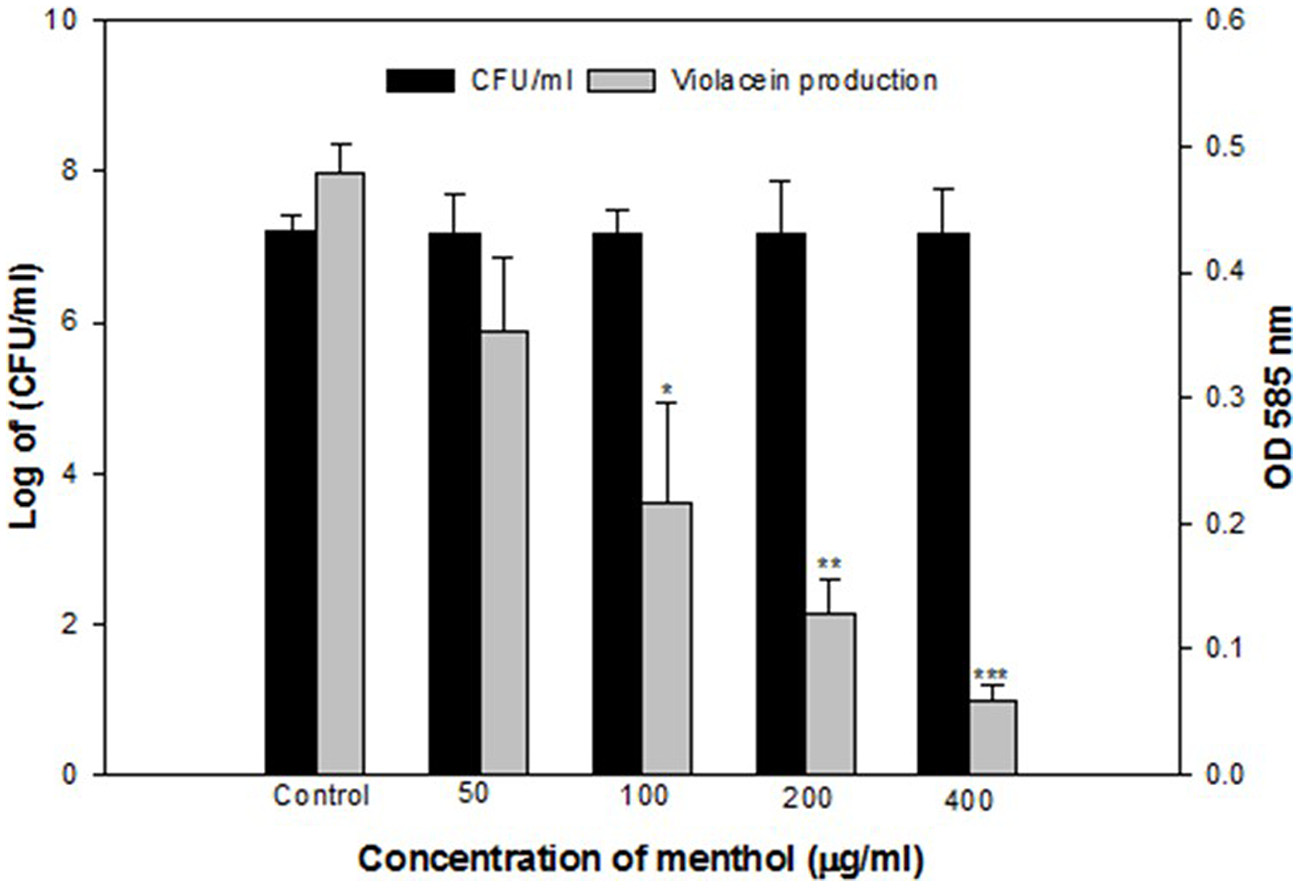
**Quantitative assessment of violacein inhibition in CVO26 by sub-MICs of menthol.** All of the data are presented as mean ± SD. Significance at ^∗^*p* ≤ 0.05, significance at ^∗∗^*p* ≤ 0.005, significance at ^∗∗∗^*p* ≤ 0.001.

Menthol exhibited a concentration dependent decrease in QS regulated violacein production. Maximum reduction of 85% was recorded at 400 μg/mLconcentration while lowest of 26% decrease over control was observed at 50 μg/mLmenthol concentration (**Figure 3**).

### Effect of Menthol on Virulence Factor Production

Effect of menthol on QS regulated virulence factors of *P. aeruginosa* PAO1 revealed a concentration dependent decrease in all the functions. Highest reduction in all the virulence factors was observed at 800 μg/mLin PAO1. Decrease in total protease activity was highest (84.2%) followed by pyocyanin production (83.5%), elastase activity (78.7%), swarming motility (78%), EPS production (57.7%), and least in chitinase activity (54.6%) as presented in **Table 4**.

**Table 4:** Effect of sub-MICs of menthol on inhibition of quorum sensing regulated virulence factors in *P. aeruginosa* PAO1.

Concentration (μg ml^-1^)	Elastase activity^a^	Total protease^b^	Pyocyanin production^c^	Chitinase activity^d^	EPS production^e^	Swarming motility^f^	Biofilm formation^g^
Control	0.141 ± 0.024	1.010 ± 0.027	5.47 ± 0.1	0.128 ± 0.015	0.997 ± 0.032	55 ± 2.7	0.677 ± 0.050
100	0.092 ± 0.015 (34.7)	0.661 ± 0.033 (34.5)	2.2 ± 0.43 (59.7)^∗^	0.109 ± 0.015 (14.8)	0.612 ± 0.038 (38.6)	41 ± 3.5 (25)	0.577 ± 0.022 (14.7)
200	0.062 ± 0.019 (56)^∗∗^	0.351 ± 0.029 (65.2)^∗∗^	2.0 ± 0.1 (63.4)^∗^	0.084 ± 0.022 (34.3)^∗^	0.581 ± 0.016 (41.7)	27 ± 4.0 (51)^∗^	0.384 ± 0.041 (43.2)^∗^
400	0.034 ± 0.012 (75.8)^∗∗∗^	0.199 ± 0.019 (80.2)^∗∗∗^	1.4 ± 0.32 (74.4)^∗∗^	0.071 ± 0.010 (44.5)^∗^	0.515 ± 0.014 (48.3)^∗^	18 ± 2.0 (67)^∗∗^	0.311 ± 0.035 (54)^∗∗^
800	0.030 ± 0.009 (78.7)^∗∗^	0.159 ± 0.019 (84.2)^∗∗∗^	0.9 ± 0.038 (83.5)^∗∗∗^	0.058 ± 0.014 (54.6)^∗^	0.421 ± 0.009 (57.7)^∗^	12 ± 3.5 (78)^∗∗^	0.207 ± 0.009 (69.4)^∗∗^

^a^Elastase activity is expressed as the absorbance at OD_495_.

^b^Total protease activity is expressed as the absorbance at OD_600._

^c^Pyocyanin concentrations were expressed as micrograms of pyocyanin produced per microgram of total protein.

^d^Chitinase activity is expressed as the absorbance at OD_570_.

^e^EPS production is expressed as absorbance at OD_480_.

^f^Swarming motility is expressed as diameter of swarm in mm.

^g^Biofilm formation is expressed as OD_470_ after incubation with crystal violet.

The data represents mean values of three independent experiments. Significance at *p ≤ 0.05, significance at **p ≤ 0.005, significance at ^∗∗∗^*p* ≤ 0.001.

Values in the parentheses indicate percent reduction over control.

In *A. hydrophila* WAF38, menthol inhibited total protease significantly (52.5%) at 200 μg mL^-1^ while at lower concentrations reduction observed was not statistically significant. EPS produced by untreated *A. hydrophila* WAF38 was lowered significantly (58.3–66.6%) at sub-MICs (50–200 μg/mL) as depicted in **Table 5**.

**Table 5:** Effect of sub-MICs of menthol on inhibition of quorum sensing regulated virulence factors in *A. hydrophila* WAF-38.

Concentration (μg ml^-1^)	Total protease^a^	EPS production^b^	Biofilm formation^c^
Control	0.854 ± 0.043	1.06 ± 0.038	0.301 ± 0.016
25	0.754 ± 0.024 (11.7)	0.698 ± 0.024 (34.1)	0.217 ± 0.031 (27.9)
50	0.636 ± 0.023 (25.5)	0.442 ± 0.010 (58.3)^∗^	0.166 ± 0.012 (44.8)^∗^
100	0.512 ± 0.034 (40.0)	0..369 ± 0.026 (65.1)^∗^	0.082 ± 0.007 (72.7)^∗∗^
200	0.405 ± 0.02 (52.5)^∗^	0.353 ± 0.018 (66.6)^∗^	0.06 ± 0.003 (80.0)^∗∗∗^

^a^Total protease activity is expressed as the absorbance at OD_600_.

^b^EPS production is expressed as absorbance at OD_480_.

^c^Biofilm formation is expressed as OD_470_ after incubation with crystal violet.

The data represents mean values of three independent experiments. Significance at ^∗^*p* ≤ 0.05, significance at **p ≤ 0.005, significance at ^∗∗∗^*p* ≤ 0.001. Values in the parentheses indicate percent reduction over control.

### Effect of Menthol on Biofilm Formation

**FIGURE 4:**
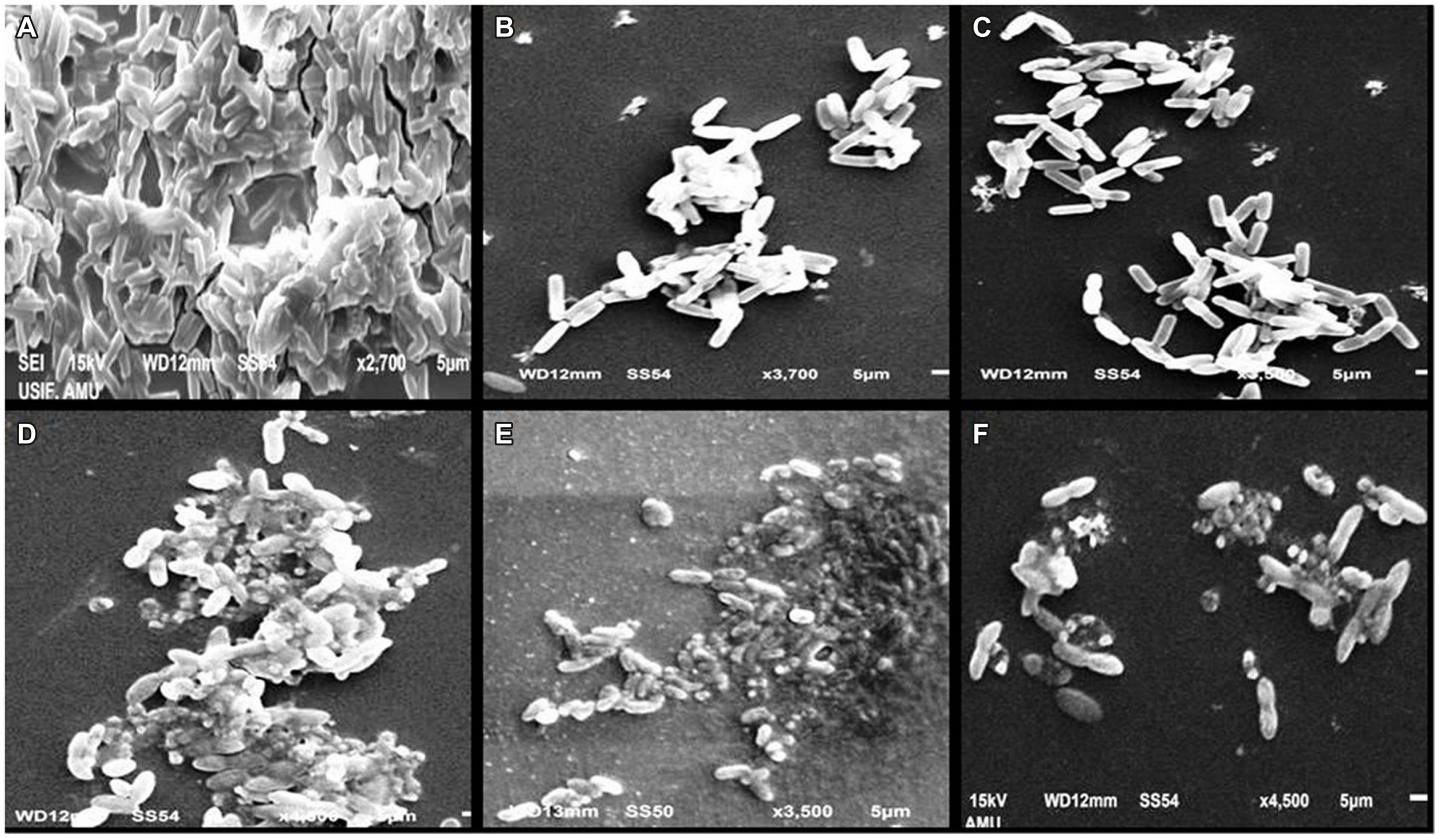
**Scanning electron microscopic (SEM) images for inhibition of biofilm of *Pseudomonas aeruginosa* PA01 at sub-MICs. (A)** Control; **(B)** PMO (3% v/v); **(C)** Menthol (800 μg mL^-1^); **(D)** *Aeromonas hydrophila* WAF38 *control*; **(E)** PMO (0.8% v/v); **(F)** menthol (200 μg mL^-1^).

A significant decrease in biofilm formation was observed in test bacterial strains when grown in the presence of menthol. Highest reduction (69.4%) in biofilm formation was observed at 800 μg/mLconcentration followed by 54 and 43.2% reduction at 400 and 200 μg/mL, respectively. To ascertain the structures visualized by light microscopy that exhibited antibiofilm activity, we used SEM to elucidate the potential of menthol against biofilm formation (**Figure 4**). The results of electron microscopic analysis revealed that the control slides showed well developed dense biofilm growth of PAO1, whereas, PAO1 treated with the menthol developed poor biofilm growth compared to that of the control sample (**Figure 4**). In *A. hydrophila* WAF38, biofilm formation was also reduced considerably ranging from 27.9–80% over untreated control at sub-MICs of menthol tested (**Table 5**).

**FIGURE 5:**
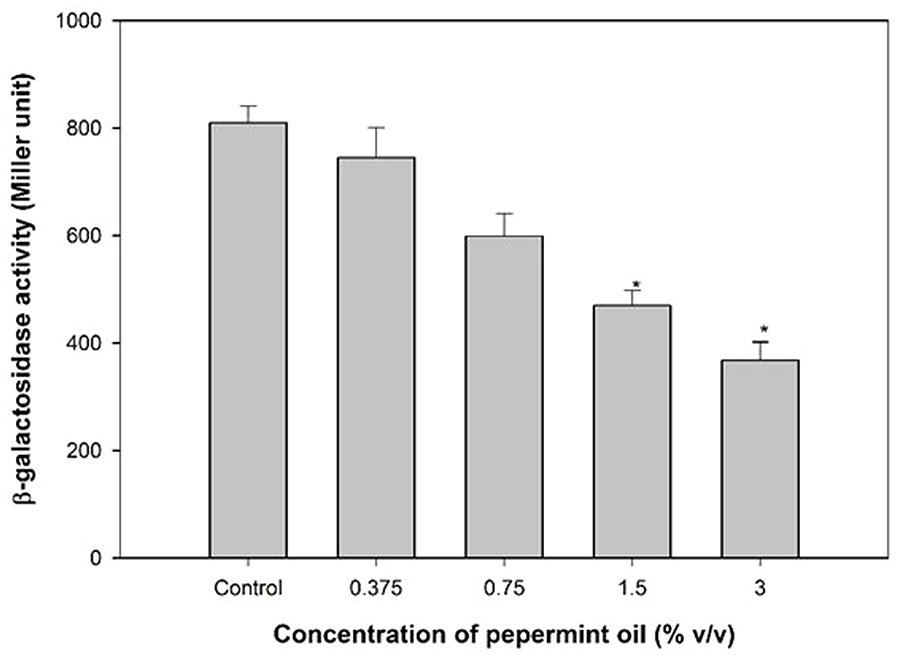
**Effect of PMO on *las* systems.** β-Galactosidase activity was measured in the *Escherichia coli* MG4/pKDT17 with and without sub-MICs of PMO. All of the data are presented as mean ± SD. Significance at ^∗^*p* ≤ 0.05.

### Effect of Menthol on *las* and *pqs* Systems

**FIGURE 6:**
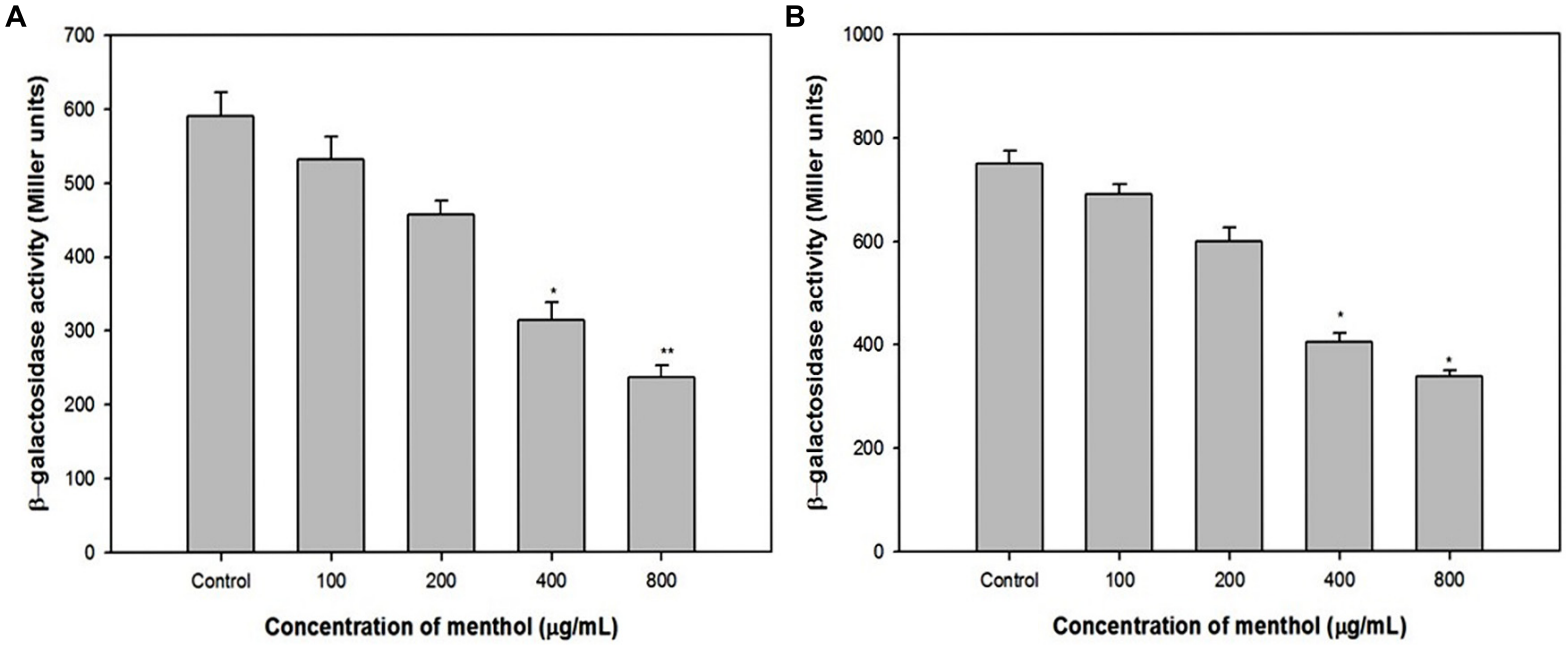
**Effect of menthol on *las* and *pqs* systems. (A)** β-Galactosidase activity was measured in the *E. coli* MG4/pKDT17 with and without sub-MICs of menthol. **(B)** β-Galactosidase activity was measured in the *E. coli* pEAL08-2 with and without menthol. All of the data are presented as mean ± SD. Significance at ^∗^*p* ≤ 0.05, significance at ^∗∗^*p* ≤ 0.005.

To exclude the influence of other QS systems in *P. aeruginosa*, we used *E. coli* MG4/pKDT17 that produces LasR and contains the *lasB* promoter fused to *lacZ*. The addition of menthol decreased β-galactosidase luminescence in *E. coli* MG4/pKDT17 up to 60% at 800 μg/mL (**Figure 6A**), which shows that menthol directly inhibits las-controlled transcription.

Pyocyanin production which is mainly regulated by the pqs system, therefore a heterologous strain *E. coli* pEAL08-2 that produces PqsR and contains the *pqsA* promoter fused to *lacZ* was used to determine whether the inhibition of pyocyanin production was directly due to the effects of pqs system (Cugini et al., 2007). The addition of menthol reduced the β-galactosidase luminescence in *E. coli* pEAL08-2 up to 55% at 800 μg/mL (**Figure 6B**), which proved that menthol directly inhibits PQS-stimulated transcription.

### *In Vivo* Assessment with *C. elegans*

**FIGURE 7:**
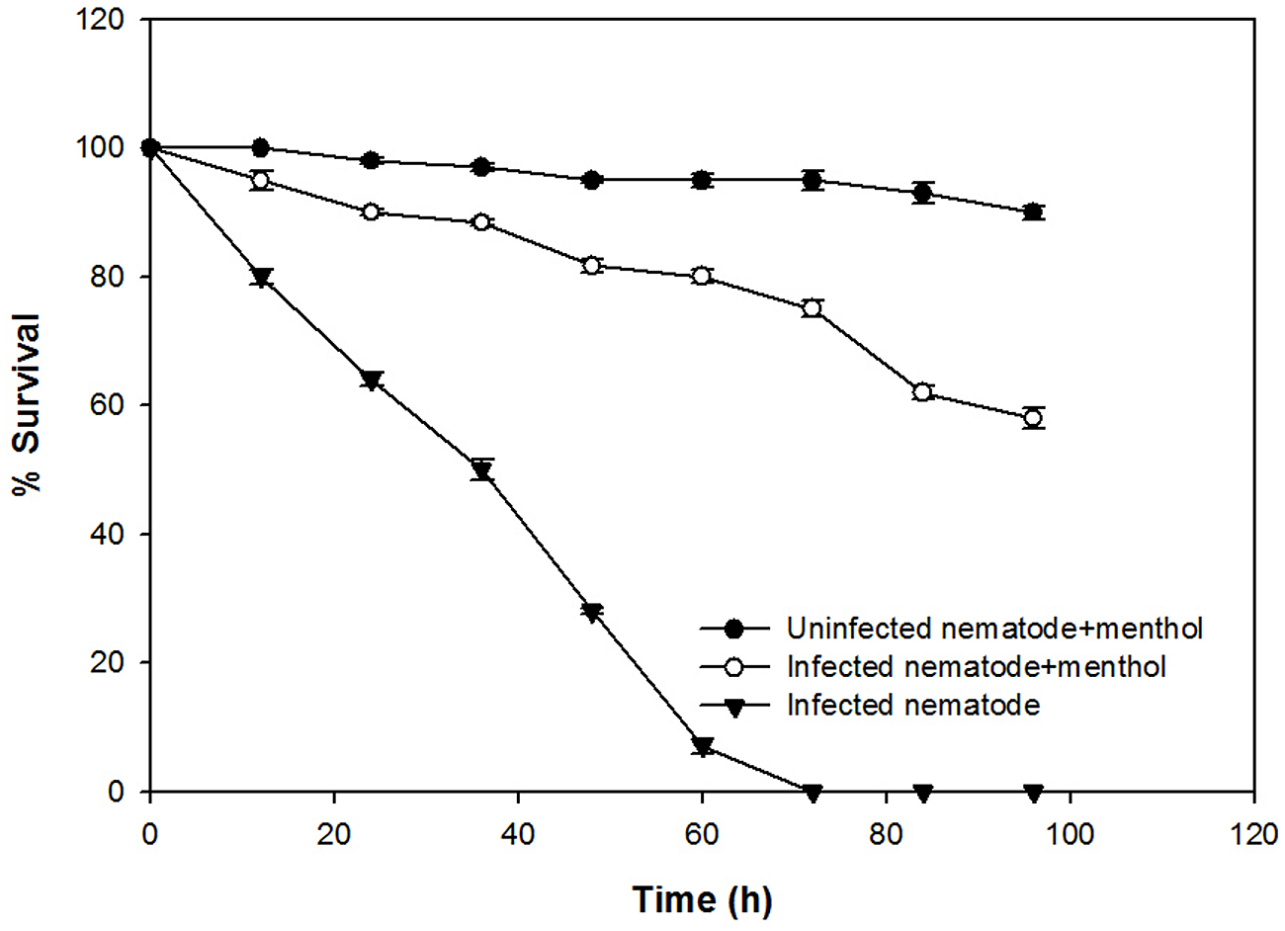
**Anti-infection potential of sub-MIC of menthol (800 μg/mL) in increasing the survival of *Caenorhabditis elegans* preinfected with *P. aeruginosa* PAO1.** Means values of triplicate independent experiments and SDs are shown.

The anti-infection potential of the sub-MIC of menthol was assessed using a liquid killing assay of *C. elegans* animal model by PAO1 in a 24-well microtitre plate. Complete mortality of the *P. aeruginosa* PAO1 preinfected *C. elegans* was observed within 72 h. However, *C. elegans* preinfected with PAO1 further treated with menthol (800 μg/mL) displayed enhanced survival upto 58% (**Figure 7**). However, menthol alone demonstrated no significant mortality of *C. elegans* at tested concentrations.

## Discussion

In the present investigation, we showed the QS inhibitory potential of PMO and menthol based on its ability to inhibit AHL-dependent violacein production in *C. violaceum* and virulence factors such as elastase, protease, pyocyanin, EPS production, and biofilm formation in PAO1. It has previously been confirmed that in *C. violaceum*, the CviIR-dependent QS system coordinates the production of violacein pigment, and the compound, which has the ability to inhibit the violacein production without any antibacterial activity, is considered to be the promising QS inhibitor (Choo et al., 2006; Zhu and Sun, 2008). In this study, a dose dependent decrease of AHL mediated violacein production was recorded in *C. violaceum* CVO26. The concentration dependent response of anti-QS potential as found in our study is also supported by the findings on clove oil (Khan et al., 2009), marjoram (Kerekes et al., 2013), and three isothiocyanates (Borges et al., 2014) that demonstrated reduction in AHL dependent violacein production in *C. violaceum* CVO26 without inhibition of growth.

The LasIR-encoded protease and elastase play a key role in the pathogenesis of PAO1 (Kessler et al., 1993). These enzymes degrade the structural components of the infected tissue and enhance the growth and invasiveness of the organism. In this present investigation, the oil of peppermint and menthol demonstrated a concentration-dependent inhibition of virulence factor production such as total protease, elastase in PAO1, as shown in **Table 1**. This result is in agreement with the study of Adonizio et al. (2008), who demonstrated significant inhibition of LasB activity by medicinal plants from Florida. In addition to this recently, flavanones (Vandeputte et al., 2011), *Lagerstroemia speciosa* fruit extract (Singh et al., 2012) and *Sclerocarya birrea* bark extract (Sarkar et al., 2014) have shown to inhibit elastase activity to substantial levels. Our data shows that menthol decreases both the elastase activity of PAO1 and the transcriptional activation of *lasB* in *E. coli*, which indicates that menthol inhibits the *las* system. Pyocyanin metabolite causes severe toxic effects by damaging the neutrophil-mediated host defense in patients with cystic fibrosis (Fothergill et al., 2007). Similarly, menthol reduced both the pyocyanin production of PAO1 and the transcriptional activation of *pqsA* in *E. coli*, which indicates that menthol inhibits the *pqs* system. Considering the *las* and *pqs* systems regulate the expression of numerous virulence-related genes, using menthol to inhibit these two systems would significantly decrease *P. aeruginosa* virulence. Our results are in accordance with the results of a recent report wherein Vandeputte et al. (2011) and Zhou et al. (2013) demonstrated that eugenol and flavanones (i.e., naringenin, eriodictyol, and taxifolin) reduced the production of pyocyanin significantly. QS-regulated flagellar and pili dependent swarming motility is considered as one of the virulence factors because of its involvement in biofilm formation (Niu and Gilbert, 2004). In the present study, the oil of pepermint and menthol reduced the swarming migration of target organism. Our findings are in agreement with the results of Khan et al. (2009) in which sub-MICs of clove oil decreased the swarming behavior in PAO1 by 78%. Recently, studies on *Capparis spinosa*, *Cuminum cyminum,* and *S. birrea* (Packiavathy et al., 2011, 2012; Sarkar et al., 2014) have shown significant reduction in swarming motility of *P. aeruginosa* PAO1.

Exopolymeric substance plays a defining role in maintaining the biofilm architecture. Therefore, decrease in production of EPS will render the biofilm structure weak and susceptible (Bomchil et al., 2003). The observed results in the present study indicated the potential PMO to inhibit EPS production in PAO1 at sub-MIC values. This is probably the first report on the inhibition of EPS by *M. piperita* oil in *P. aeruginosa* PAO1. Formation of biofilm by PAO1 is QS regulated and this mode of growth confers increased drug resistance and infection caused is quite severe in patients suffering from cystic fibrosis. AHL mediated cell–cell signaling plays a crucial role in the initiation and maturation of biofilms in PAO1 (Rasmussen et al., 2005). The results of our present investigation revealed the biofilm inhibiting potential of PMO and its major phytoconstituent menthol against PAO1 biofilms in a concentration dependent manner, as shown in **Figure 3**. Recent studies on herbal extract of *M. piperita* have demonstrated 57% reduction in adhesion property of *P. aeruginosa* (Sandasi et al., 2011); our findings on the biofilm inhibitory property of *M. piperita* oil finds support from above work. Further, the results obtained are comparable to the decrease in biofilm formation reported in studies for methyl eugenol (Packiavathy et al., 2012), marjoram (Kerekes et al., 2013), and *Rosa rugosa* tea extract (Zhang et al., 2014).

The mortality of the nematode by PAO1 is caused by the cyanide asphyxiation and paralysis (Gallagher and Manoil, 2001). We demonstrated an enhanced survival of pre-infected nematode model maintained with menthol (800 μg/mL) as depicted in the **Figure 7**. The data obtained clearly indicates that the menthol interferes with the QS of PAO1 leading to reduced mortality of the *C. elegans*. The findings are similar to the previous observations of Husain et al. (2013), on enhanced survival of the nematode (*C. elegans*) after treatment with sub-MICs of clove oil.

## Conclusion

In many pathogens, virulence potential and biofilm formation is under the regulation of QS. Therefore, instead of bactericidal or bacteriostatic strategies, attenuation of virulence via QS inhibition approach could be more effective strategy to combat bacterial infection caused by drug resistant bacteria.. In the present study oil of *M. piperita* and menthol showed QS inhibitory properties, resulting in decreased biofilm formation capabilities and the attenuation of *P. aeruginosa* virulence *in vitro* and *in vivo*. Findings of this study also suggest that essential oil of *M. piperita* and menthol can inhibit QS, biofilm and its related virulence processes in pathogenic bacteria, and can be exploited by the pharmaceutical industry for the development of new safe broad spectrum antibiofilm and anti-QS drugs with reduced toxicity and antibiotic resistance.

## Conflict of Interest Statement

The authors declare that the research was conducted in the absence of any commercial or financial relationships that could be construed as a potential conflict of interest.
