# Supplementary material for: Sub-MICs of Mentha piperita essential oil and menthol inhibits AHL mediated quorum sensing and biofilm of Gram-negative bacteria
Source: Front Microbiol. 2015 May 13;6:420. doi: 10.3389/fmicb.2015.00420 (PMC4429619; doi:10.3389/fmicb.2015.00420)
Supplement: Supplementary file 1 [file Table_1.PDF]

Supplementary Material

Sub-MICs of *Mentha piperita* essential oil and menthol inhibits AHL mediated quorum sensing and biofilm of Gram negative bacteria

Fohad Mabood Husain[a, b*], Iqbal Ahmad[a*], Mohammad Shavez Khan[a], Ejaz Ahmad[c] , Qudisa Tahseen[d], Mohammad Shahnawaz Khan[e], Nasser A. Alshabib[b]

[a]Department of Agricultural Microbiology, Aligarh Muslim University, Aligarh-202002, UP, INDIA
[b]Department of Food Science and Nutrition, College of Food and Agricultural Sciences, King Saud University, Riyadh-11541, Kingdom of Saudi Arabia
[c]School of Pharmaceutical Sciences, Sao Paulo State University- UNESP, Araraquara, Sao Paulo – Brazil, 14.801-902
[d]Department of Zoology, Aligarh Muslim University, Aligarh-202002, UP, INDIA
[d]Department of Biochemistry, College of Science, King Saud University, Riyadh-11541, Kingdom of Saudi Arabia

Corresponding authors*

Dr. Iqbal Ahmad
Department of Agricultural Microbiology, Aligarh Muslim University, Aligarh-202002-UP, INDIA; Phone: +91 9897902936; Fax: +91 571 2703516; E-mail: ahmadiqbal8@yahoo.co.in

Dr. Fohad Mabood Husain
Department of Food Science and Nutrition, College of Food and Agricultural Sciences,
King Saud University, Riyadh-11541, KSA
Phone: +966599306733; E-mail: fahadamu@gmail.com

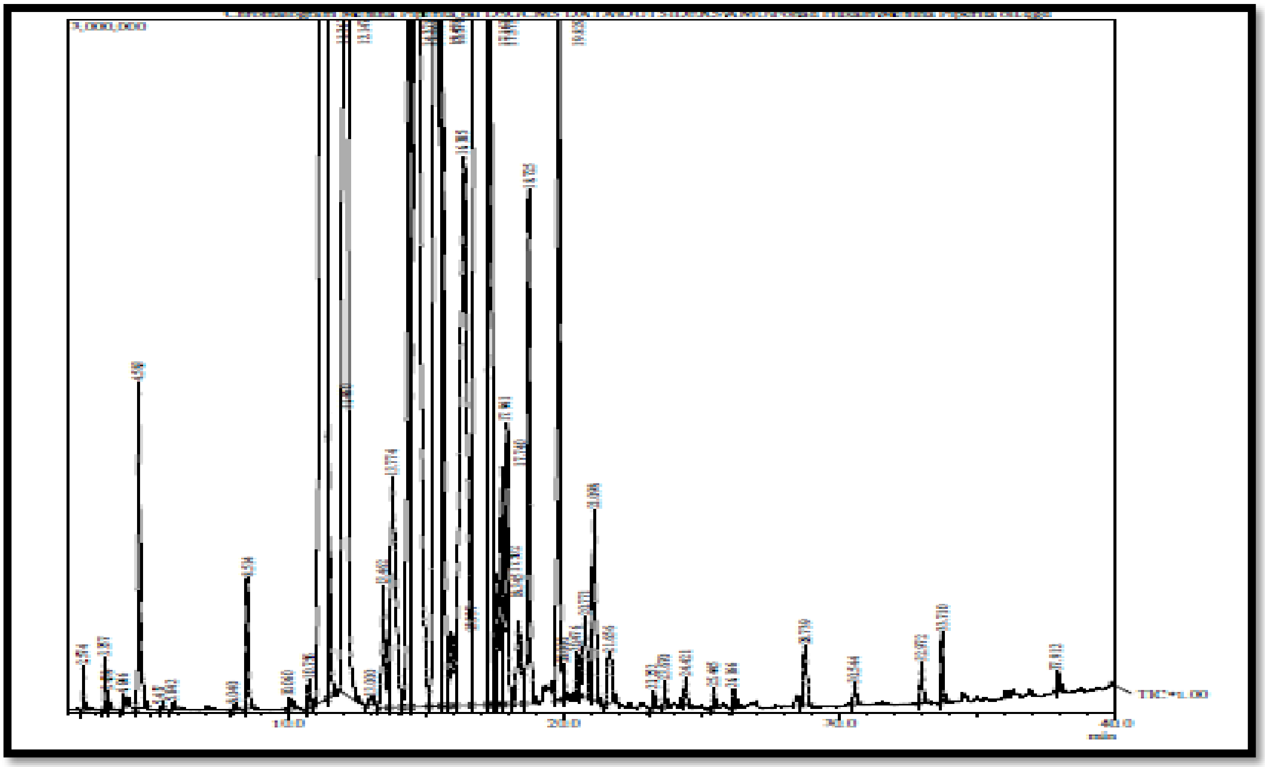

Fig. S1 GC-MS chromatogram of *Mentha piperita* (peppermint) oil

Table S1 Components of peppermint (*Mentha piperita*) essential oil as identified by GC-MS analysis.

Peak no.	Components	Retention time	Area (%)
1.	Limonene	4.58	0.53
2.	Menthone	11.36	16.44
3.	Isomenthone	12.14	10.47
4.	1-Hydroxyoctane	13.77	0.84
5.	Isopulegol	14.37	2.16
6.	Menthyl acetate	14.70	7.47
7.	Neoisomenthol	15.43	11.33
8.	Neoisopulegol	15.57	1.84
9.	2-Isopropyl-5-methylcyclohexanol	16.38	2.74
10.	Menthol	17.16	36.87
11.	Pulegone	17.37	1.60
12.	Lavandulol	17.94	0.56
13.	α-Terpineol	18.72	0.99
14.	Piperitone	19.82	2.17
